# DetR DB: A Database of Ionizing Radiation Resistance Determinants

**DOI:** 10.3390/genes11121477

**Published:** 2020-12-09

**Authors:** Alina Ryabova, Olga Kozlova, Azat Kadirov, Anastasiia Ananeva, Oleg Gusev, Elena Shagimardanova

**Affiliations:** 1 Institute of Fundamental Medicine and Biology, Kazan Federal University, Kazan 420008, Russia; urban-nomad@yandex.ru (A.R.); olga-sphinx@yandex.ru (O.K.); bilinet@mail.ru (A.K.); nastya.ananeva@gmail.com (A.A.); oleg.gusev@riken.jp (O.G.); 2 Laboratory for Transcriptome Technology, RIKEN Center for Integrative Medical Sciences, RIKEN, Yokohama 230-0045, Japan

**Keywords:** ionizing radiation, radiation resistance, genetic mechanisms, microorganisms

## Abstract

Nuclear pollution is an urgent environmental issue and is a consequence of rapid industrialization and nuclear accidents in the past. Remediation of nuclear polluted sites using microbial vital activity (bioremediation) is a promising approach to recover contaminated areas in an environmentally friendly and cost-saving way. At the same time, the number of known bacterial and archaeal species able to withstand extremely high doses of ionizing radiation (IR) is steadily growing every year, together with growing knowledge about mechanisms of radioresistance that opens up opportunities for developing new biotechnological solutions. However, these data are often not systemized, and can be difficult to access. Here, we present the Determinants of Radioresistance Database, or DetR DB, gathering a comprehensive catalog of radioresistant microbes and their molecular and genetic determinants of enhanced IR tolerance. The database provides search tools, including taxonomy, common gene name, and BLAST. DetR DB will be a useful tool for the research community by facilitating the extraction of the necessary information to help further analysis of radiation-resistant mechanisms.

## 1. Introduction

Ionizing radiation (IR) is widely present in the daily lives of all organisms, originating from natural and manmade sources. Environmental IR or natural background radiation is largely from cosmic rays from deep space and the naturally radioactive elements that are relatively abundant in rocks and soils that can be found in water, vegetation, and food. Background radiation levels vary geographically due to geological differences, although mostly at low levels. Artificial IR comes from human-made sources ranging from nuclear fuel cycles and industry to medical applications for diagnosis and treatment [1,2].

It is worth mentioning separately that IR exposure from radioactive pollution may be extremely hazardous. It occurs when there is a presence or depositions of radioactive substances, especially as a result of nuclear accidents, acts of military aggression, or conducting nuclear tests [3]. Nuclear fallouts in Hiroshima and Nagasaki (1945) in the Second World War and a number of tragic nuclear power plant accidents like the Three Mile Island accident (1979), Chernobyl (1986), and the Fukushima Daiichi (2011) nuclear disaster that left many instantly dead and caused severe damage to the environment for many more years by the radiation released [4]. Radioactive contamination is a serious threat to almost all life forms, since radioactive pollution maintains its radioactive properties for decades to millennia [2].

Radiation-induced damage is determined by parameters such as the type and dosage of IR rays, length of exposure, and, most importantly, the characteristics of the exposed individual [5]. An exceptional ability to withstand the lethal effects of IR is widespread among organisms that inhabit extreme environments. These organisms, known as extremophiles, are a largely unexplored group that survive in habitats that are hostile or even deadly for other life forms [6]. Extremophiles are found in all domains of life, but the vast majority of them belong to Bacteria and Archaea species. Such extremophilic microorganisms are highly adapted to thrive in natural conditions and can thrive in broad ranges of temperature, salinity, pH, water, and nutrient limitation. This is the norm in which they are able to metabolically and biochemically operate. In addition to natural habitats that can be defined as “extreme” based on anthropocentric criteria, many independent scientific studies have demonstrated life to be present in artificial environments with adverse factors for living beings, even high levels of IR. Thus, some extremophilic bacterial and archaeal species have been isolated from high-level radioactive waste sites at Savannah River in South Carolina and at Hanford in Washington [7,8], spent nuclear fuel storage pools at Sellafield, UK [9], Madras Atomic Power Station, Kalpakkam [10], German salt dome Gorleben [11], and the Chernobyl Nuclear Power Plant buildings [12,13,14]. According to the most widely accepted theory, the origin of cross-resistance to IR and other types of stress may be related to overlap between mechanisms of resistance to IR and other stressors [15]. However, radioresistance is not limited to extremophiles but can occur among common microbial species, as it was found in river sediment soils exposed to radioactive contamination from Fukushima [16], or it can be cultivated in a laboratory from radiosensitive variants [17].

Radiation resistant microorganisms possess a huge potential for biotechnological applications in the field of bioremediation of radioactive waste in an eco-friendly and cost-saving way. To date, the most developed bioremediation method based on the microbial usage employs the widely known radiation-resistant bacterium *Deinococcus radiodurans* [18], although research is being done on the possible use of other microorganisms for these purposes [19,20,21,22,23]. On the other hand, in addition to the use of such microbes in the disposal of radioactive waste, it is unclear how radioresistant microbes affect the structural materials of nuclear waste storage facilities and the environment of nuclear reactors, and whether they can lead to their biodegradation. Resistance to high doses of IR is obviously a hot topic, but despite that, there is not much organized up-to-date information about radioresistant organisms and their mechanisms of radioresistance.

In order to combine and systematize rapidly accumulating knowledge about organisms that are able to thrive in conditions of high levels of IR, we designed the Determinants of Radioresistance Database (DetR DB, http://extremebiolab.com/detr-db/). This is an open-access online tool based on peer-reviewed articles of radioresistant microorganisms with an emphasis on their known molecular and genetic benefits that determine enhanced IR tolerance. The current version of the database contains 63 species of the Bacteria and Archaea domains and a description of about 4000 genes. Users can search DetR DB using several fields, including taxonomy or BLASTn/BLASTp. We hope that DetR DB can make it easier for researchers to get the necessary data and help in the early development of effective bioremediation methods.

## 2. Materials and Methods

### 2.1. Source of Data

The DetR DB compiles both manually curated information and automatically generated data that is regularly managed and updated. We analyzed published literature available in the PubMed database, covering all reports of isolation and identification of radioresistant microorganisms. The initial search for articles was performed using keywords such as ionizing radiation, radiation resistance, genetic mechanisms, microorganisms, and combinations of these keywords. Eventually, 132 articles were selected, with preference given to studies that specified a D10 value (D10 threshold is the absorbed radiation dose required to inactivate 90% of a viable population). If the D10 value was not yet known, however, and the bacterial or archaeal species belonging to genera with several other known radioresistant organisms (like *Deinococcus cellulosilyticus*) or species isolated from extreme environments (for example, *Pyrococcus horikoshii*, which was first isolated from hydrothermal fluid samples), such species were also included to the database. As a result, 63 species of microorganisms were selected, and the D10 value was determined experimentally for 42 of them. The same procedure was used to search for genes, proteins, or pathways involved in the formation and functioning of mechanisms that determine the resistance to IR. As with the selection process of most microbial species, we focused on those studies where the role of the specific determinant was confirmed experimentally. Thus, the database currently contains 119 genes of interest.

### 2.2. Database Construction

Each strain of selected microorganism was matched with the corresponding UniProt ID number that was used to obtain and bind diverse data on species and genes. Taxonomy of radioresistant microbes was obtained from the NCBI database. The metadata on genes and proteins, including amino acid sequences, were gathered from UniProt. Nucleotide sequences and gene ontology (GO) annotations were downloaded from the KEGG genes database. The final database was localized on the web server (http://extremebiolab.com/detr-db/) and added as Supplementary Materials Tables S1–S3. This software was written in C# with .NET Framework 4 platform usage. Windows Presentation Foundation (WPF) was used to build the user interface. We used PHP to build the web page and get user requested information from database, which is simply stored on the server as CSV-table.

## 3. Results

**Figure 1:**
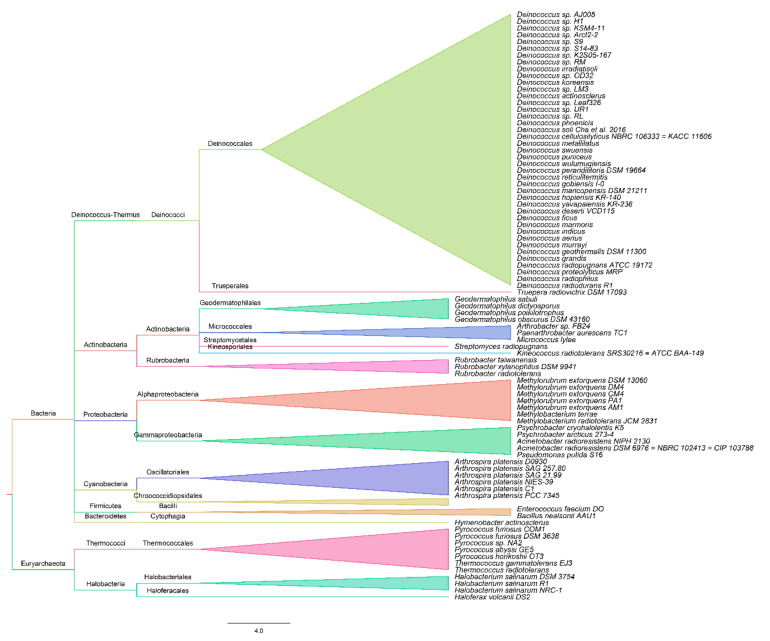
Phylogenetic tree containing microbial species included in the Determinants of Radioresistance Database (DetR DB).

The DetR DB is exclusively focused on relevant information about genetic and molecular determinants of enhanced radioresistance in a species-specific manner. At the time of writing, the database includes 55 species of the Bacteria domain and 8 species of the Archaea domain (Figure 1).

**Figure 2:**
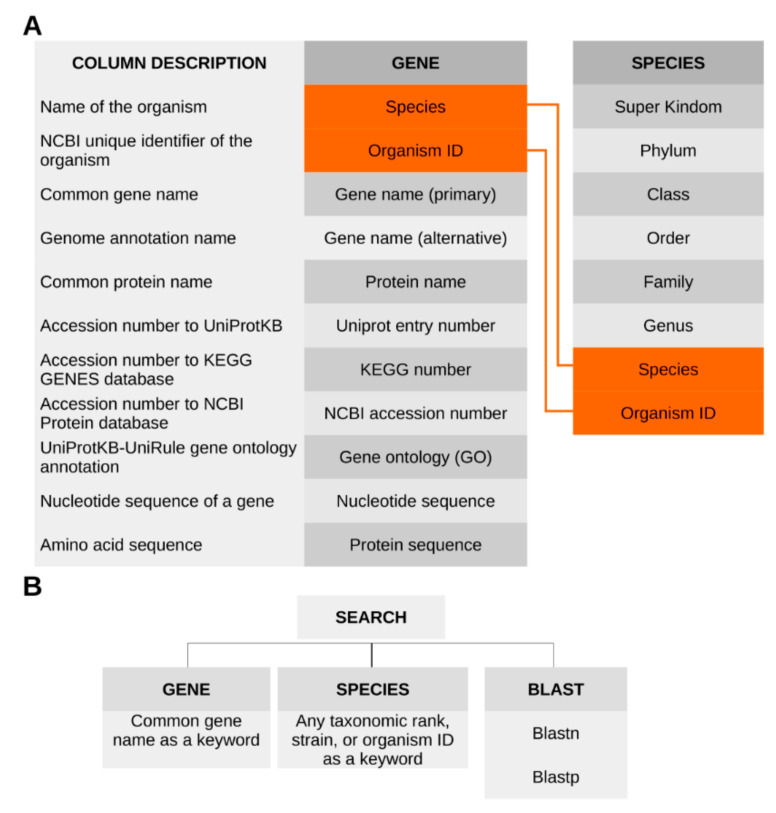
Structure and search tools of the DetR DB. (**A**) Structure and content of the database. Orange boxes indicate the interlinked columns. The column on the left explains different attributes of a “GENE” entity. (**B**) There are three options for search: by gene name, by taxonomy, or with BLAST tool.

The content of the database is organized into three sections that contain BLAST search, taxonomy of microorganisms, and gene annotation (Figure 2).

The last two sections are interconnected via specific links. The taxonomy section provides a description of the taxonomic lineage of microorganism, including the main nomenclature ranks from domain to strain, as well as the organism ID number (UniProt). The gene annotation section contains metadata that defines the species or strain with its organism ID number and its determinant: primary gene name, alternative gene name, protein name, UniProt entry number, KEGG number, NCBI accession number, GO, nucleotide sequence, and protein sequence. There are two search modes for these sections: by taxonomy and by gene name. The taxonomy search can be carried out by entering any taxonomic rank, strain name, or organism ID. The gene name search, as the title suggests, allows making a query through the common gene name as a keyword. The database can be searched with sequence-based queries by the standard BLAST tool that will compare a user-submitted sequence against nucleotide or amino acid sequence records in DetR DB.

**Figure 3:**
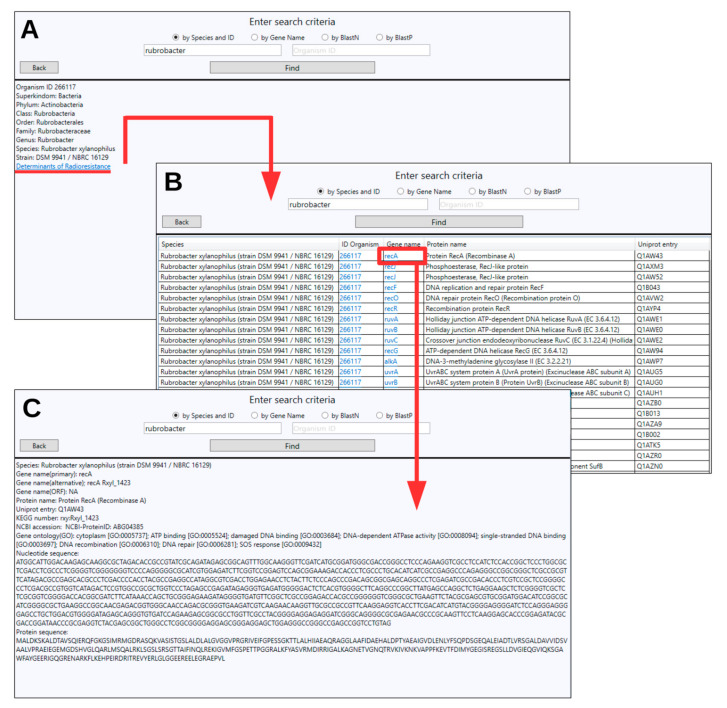
An example of search and navigation in the DetR DB. (**A**) The organism description page provides organism ID, taxonomy, and the link to the list of all determinants of ionizing radiation resistance for this species that are contained in the database. (**B**) The list of genes related to enhanced radioresistance of particular microorganisms, including common gene name, protein name, and UniProt entry number. (**C**) From the list of radioresistance determinants, the user can select a gene of interest and go to the page with its detailed description.

As a user case of the database operation, an example of searching by the keyword “rubrobacter” can be provided (Figure 3), where the user can select the concrete species and the strain of interest from the available list (these steps are not shown in the figure) and go to the page with its description (Figure 3A). In addition to the taxonomy, the species description page allows one to go to a list of all determinants of radioresistance available in the database for this strain (Figure 3B). After selecting a gene of interest, the user goes to a page with its detailed description, which includes nucleotide and amino acid sequences that can later be used for BLAST search (Figure 3C).

## 4. Discussion

DetR DB will continue to grow with the inclusion of new published data and adding more details to existing entries. We intend to incorporate more radioresistant bacterial and archaeal species from studies that we may have overlooked, as well as from future studies. Expanding the database with eukaryotic organisms also could be of great interest and practical use, since a large number of extremely ionizing radiation-resistant organisms are known among fungi species and even several invertebrates. Besides, DetR DB continues to make improvements to its operational stability and user interface. The visual component is planned to be given a more attractive appearance, while the interface will be provided with advanced navigation features. We continue to work on providing high-quality and up-to-date data on radioresistant organisms and their mechanisms of endurance to IR. We believe that the DetR DB could benefit a wide range of specialists, both those who are engaged in basic research and those who develop biotechnological approaches. Therefore, the DetR DB can become an efficient platform for evolutionary studies or developing synthetic biology applications that may eventually lead to creating environmentally friendly bioremediation technologies.

## Supplementary Materials

The following are available online at https://www.mdpi.com/2073-4425/11/12/1477/s1, Table S1: taxonomic lineage of radioresistant microorganisms. CSV file with taxonomic classification of microorganisms included in Det DR database, Table S2: Genetic determinants of microorganisms resistant to ionizing radiation. CSV file with Species, Organism ID, Gene name (primary and alternative), ORF, Protein name, UniProt entry, KEGG number, NCBI accession number, Gene ontology, Nucleotide sequence and Protein sequence, Table S3: references list with first report of radioresistant microorganisms included in the Det DR database. CSV file with cited literature and D10 value.
